# Recurrent activity in neuronal avalanches

**DOI:** 10.1038/s41598-023-31851-x

**Published:** 2023-03-24

**Authors:** Tyler Salners, Karina E. Avila, Benjamin Nicholson, Christopher R. Myers, John Beggs, Karin A. Dahmen

**Affiliations:** 1 Department of Physics, University of Illinois at Urbana-Champaign, 1110 West Green Street, Urbana, IL 61801 USA; 2 Physics Department, University Kaiserslautern, Erwin-Schrödinger-Straße, 67663 Kaiserslautern, Germany; 3 Laboratory of Atomic and Solid State Physics, Clark Hall, Cornell University, Ithaca, NY 14853-2501 USA; 4 Center for Advanced Computing, Cornell University, Ithaca, NY 14853 USA; 5 Department of Physics, Indiana University, Bloomington, IN 47405 USA

**Keywords:** Biological physics, Dynamical systems, Phase transitions and critical phenomena

## Abstract

A new statistical analysis of large neuronal avalanches observed in mouse and rat brain tissues reveals a substantial degree of recurrent activity and cyclic patterns of activation not seen in smaller avalanches. To explain these observations, we adapted a model of structural weakening in materials. In this model, dynamical weakening of neuron firing thresholds closely replicates experimental avalanche size distributions, firing number distributions, and patterns of cyclic activity. This agreement between model and data suggests that a mechanism like dynamical weakening plays a key role in recurrent activity found in large neuronal avalanches. We expect these results to illuminate the causes and dynamics of large avalanches, like those seen in seizures.

## Introduction

Neuronal firing signals measured from the cortex of animals and humans are known to occur in quick intermittent bursts, involving complex cascades of electrical activity^1–12^. Here we show that while the smaller cascades typically are not recurrent and occur temporally clustered, the largest cascades or avalanches are periodically occurring and highly recurrent. Understanding the origin of large avalanches and predicting their timing is important for preventing their potentially catastrophic impact for instance during a seizure. The hope is that understanding the underlying mechanism provides new insights for preventing serious problems such as epilepsy, which affects roughly one percent of the world’s population. Possible mechanisms include pre-existing feedback loops in the underlying neuronal network, temporary strengthening of active connections between neurons, or firing threshold weakening. Here we appeal to a biological threshold weakening mechanism that has been reported^13^. We find that including this mechanism in an established model for firing avalanches qualitatively reproduces the experimental data. We also discuss connections to other models.

The results of this study are relevant not just for neurons but also for many other systems: similar collective bursts of cascading activity—or “avalanches” occur in a broad range of experimental and natural systems, including magnets, fluids in porous media, and earthquakes^14–16^. The power law scaling behavior of avalanche statistics and dynamics can often be described in the framework of second order phase transitions^16^. Since the smaller avalanches in healthy brain tissue are often also power-law distributed, the brain has been conjectured to operate near a critical point^1^. In this work, we expect the weakening parameter to increase the avalanche size scaling exponent from 1/2 to 1, describing the universality class of de-pinning with dynamical weakening^14^. We will refer to these avalanches in the power-law scaling regime of the size distribution as “small avalanches”, and those that are larger than the scaling regime (i.e., in the exponential tail of the distribution) as “large avalanches”. Several statistical and dynamical model predictions have been investigated through experiments^9^, but the interplay between the dynamically changing neural network and the avalanches and especially the dynamics of catastrophically large avalanches, have yet to be fully understood.

Electrical activity in neurons travels along networks defined by neuronal connectivity. Broadly speaking, activity can propagate through a network of interconnected neuronal nodes in either a feedforward or in a feedback manner. In feedforward propagation, activity visits each neuron only once in a given cascade. Feedforward cascades are relatively brief in duration, typically stable, and can be used to perform computations that rapidly converge^17^. In feedback propagation, some nodes are reactivated, possibly many times, during a cascade. Feedback cascades can be relatively long in duration, can amplify and become unstable, and can be used to perform iterative computations that take longer to converge^18^. This type of propagation could easily be harmful as well, since it can promote self-generated activity that may not be desirable. Some experiments have found recurrent activity in neurons^19–23^, usually as the driving force behind self-generated sustained activity, where it was seen to arise during seizures in rats^20^. It was also shown to generate slow oscillations in cat neocortex^19^ and suggested to play a role in synchronization in Guinea pig hippocampal slices^21^. Despite this past work, limitations of the spatial and/or temporal resolution of most methods for recording the activity of a sufficiently large number of neurons has prevented recurrent activity within neuronal avalanches from being experimentally resolved. Here we leverage a novel technique^24^ to reexamine neuronal firing data^9,25^, allowing us to disentangle neuronal avalanches into causally connected webs of firing activity. This affords us a high enough spatial and temporal resolution to reliably (see Supplemental Material, Sec. B) characterize signatures of recurrent activity for the first time within avalanches, both qualitatively and quantitatively.

We note here that firing frequency adaptation^26^ is normally thought to suppress recurrent firing (within the timescale T). However, the recurrent firing seen in the data suggests additional mechanisms are at play. A mechanism that weakens thresholds dynamically (and temporarily) is known to affect the dynamics of many systems such as earthquakes^27^, shear deformation in materials^28^ and even the formation of river basins^29,30^. Here we invoke an analogous neuronal weakening mechanism for three main reasons: (1) Inhibitory neurons have a regime where their firing thresholds decrease as they are driven more strongly^13^. In addition, the hyperpolarizing effects of inhibition have been shown to lower firing thresholds and reduce refractory periods of excitatory neurons^31^. (2) The effects of a weakening mechanism are equivalent to the effects of temporary coupling strength increase immediately after a firing event^14^. Neuronal data support this idea as synapses onto inhibitory neurons in the cortex have short-term facilitation^32^. Thus, the literature suggests a biological tuning parameter that controls recurrent (such as “epileptic”^20^) activity in neuronal networks. The new parameter could be either temporary threshold weakening or, equivalently, a coupling strength increase. (3) Here we use the language of weakening simply to connect the description to a much broader class of systems with similar recurrence effects, including earthquakes and brittle behavior, where weakening is the more natural description. Yet, both mechanisms equally promote recurrent activity within large avalanches because they are analogous to each other, and both interpretations can be used interchangeably.

## Methods

### Data from microelectrode arrays

The data that we analyze here were collected using a 512-microelectrode array and previously published^9,25^. This array has an electrode spacing 60 μm and sampling rate (20 kHz) that let us detect individual neuron firing events and track their transmission within a densely connected cortical network. The tissue we used in these experiments came from slices of mouse and rat cortex that were cultured for two weeks until they showed mature patterns of neuronal activity. These patterns allow for extraction of avalanche size and avalanche duration distributions. For several of these samples, those distributions can be approximated by power laws, indicating that these networks may be operating near a critical point^4^. We thus focused our attention on those samples that reflect the most relevant state seen in-vivo for active and anesthetized animals^33–35^.

### Causal webs

**Figure 1:**
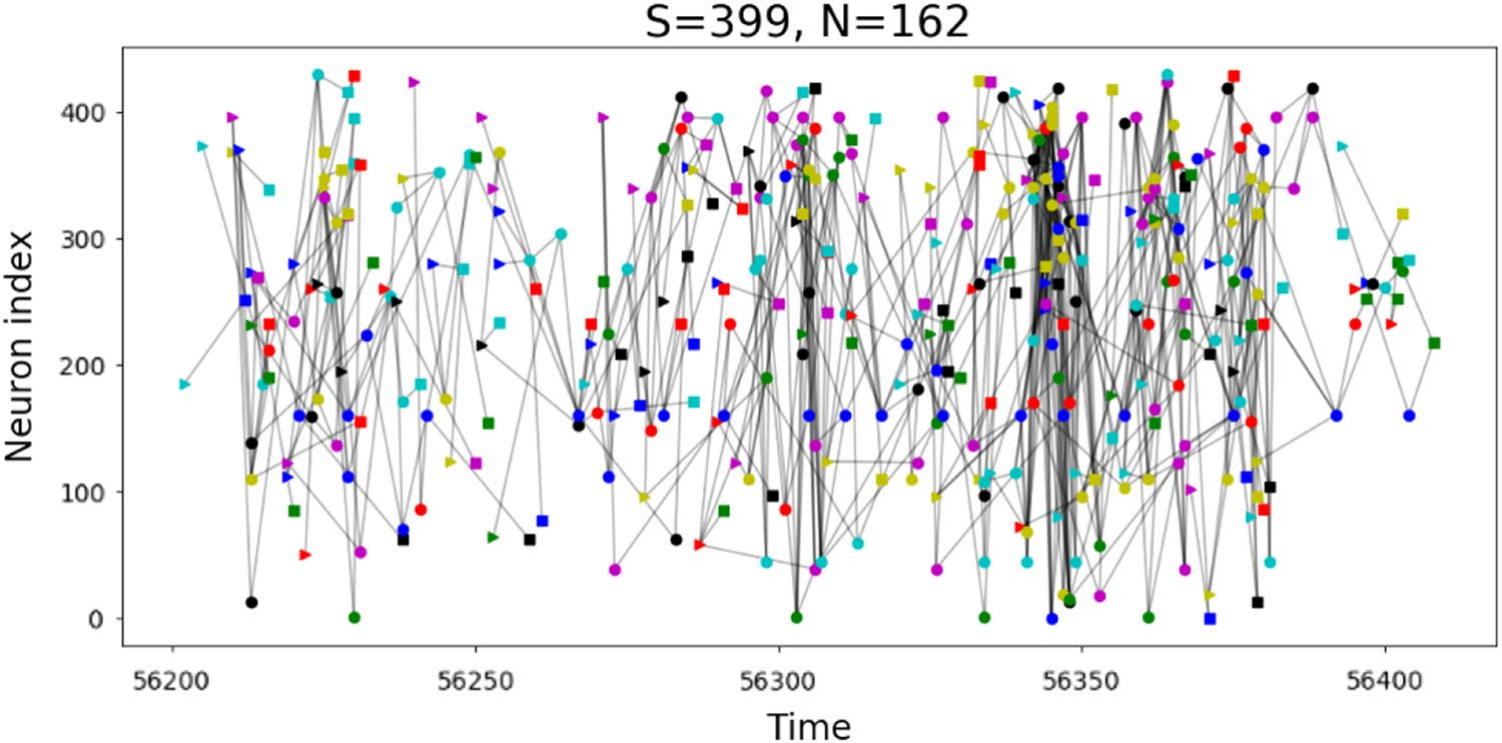
Causal webs. Time trace of a recurrent avalanche in mouse. For a single large avalanche with size S = 399 and number of neurons N = 162, the causal web of firing activity. Individual neurons are labeled by an arbitrary neuron index (y-axis, not all neurons participate in this avalanche), which fire at particular times (x-axis, in ms since start of recording). The causal web is a directed graph whose nodes are indexed by the pair (neuron, time). Nodes with no incoming edges are indicated by right-pointing triangles, nodes with no outgoing edges are indicated by squares, and nodes with both incoming and outgoing edges are indicated by circles. Patterns of recurrent neural activity are seen as particular neurons fire repeatedly during the avalanche, leading to horizontal trace of like-colored symbols.

From firing activity, we need to infer neuronal avalanches. Most previous work has relied on temporal binning of the data, to identify temporally connected bins of activity^1^. One problem with this method is that it can merge avalanches that occur at the same time but at disconnected locations. In this work, we have used instead a recently developed method of “causal webs”^24^. Briefly, this assumes that avalanches can only propagate along connections between neurons and at delays that are consistent with those connections. We used transfer entropy^36^ to estimate statistically significant functional connections between neurons and their corresponding delays^37–40^. We then mapped the observed network activity onto avalanches that were consistent with the connections and delays we found previously in the network. Activity that could not be accounted for by these connections and delays was excluded from avalanches. From the raw firing data, we extracted causal webs for several mouse and rat neural tissue recordings (a single avalanche is shown in Fig. 1). Generally, each recording consisted of many tens to hundreds of thousands of avalanches involving hundreds of neurons. Not all samples show evidence of power-law distributed avalanche statistics, so we use such statistics as a quality control filter, resulting in six mouse samples and two rat samples which we use for further analysis (see supp, Fig. S1).

### Bi-test

The Bi-test extracts temporal information about avalanches in the form of a distribution of interevent times^41^. These times can be broadly classified as either clustered or periodic: information that can suggest different mechanisms underlying recurrent avalanche activity. Given a set of *N* avalanches one can observe the set of starting times $T={t_\text {i}}, i = 1, \ldots ,N$ to construct the values $H_{i}=\Delta t_\text {i}/(\Delta t_\text {i}+\Delta \tau _{i}/2)$. Here $\Delta t_\text {i}$ is taken as the minimal interevent time, $\text {min}(|t_\text {i}-t_\text {i-1}|,|t_\text {i+1}-t_\text {i}|)$, and $\Delta \tau _\text {i}$ is the subsequent interevent time $(|t_\text {i-1}-t_\text {i-2}|$ or $|t_\text {i+2}-t_\text {i+1}|)$. Once $H=\{H_\text {i} \}$ is found, the form of the temporal correlations can be inferred from the shape of the cumulative distribution function (CDF) of *H* (see Fig. S13)^41,42^.

### Model

The broad model describing systems like earthquakes and magnets^14–16^ is adapted to include not only excitatory (i.e. positively coupled), but inhibitory (i.e. negatively coupled) cells as well, to better represent real neuronal systems. We further replace the uniform coupling constant J by a weight matrix $J_\text {ij}$ with an all-to-all topology and the strengths randomly distributed. This represents the connections between a pre ($\text {i}$) and post ($\text {j}$) synaptic neurons. In view of experimentally observed functional connectivity^39,43,44^, the elements of this matrix are sampled from a log-normal distribution

1 $$\begin{aligned} P(J_\text {ij})=\frac{1}{J_\text {ij}\sqrt{2 \pi N_\text {t}}} e^{\frac{-\ln (J_\text {ij})^2}{2N_\text {t}}} \end{aligned}$$

We note that $J_{\textrm{ij}}$ is not necessarily the same as $J_{\textrm{ji}}$. Simulations start by defining for all neurons an arrest potential $V_\text {i,arrest}$ that is sampled from a uniform distribution on the interval $[-w/2,w/2]$. We initialize all $N_\text {t}$ neurons with a random potential value

2 $$\begin{aligned} V_\text {i} = V_\text {i,arrest} +\xi \Delta V_\text {i} \end{aligned}$$

where $0 \le \xi \le 1$ is uniformly distributed and for simplicity we have defined $\Delta V_\text {i} =V_\text {thresh} - V_\text {i,arrest}$. This simply initializes the system in a mixed up state so the steady state is reached quickly. Next, the inclusion of inhibitory neurons amounts to designating $N_\text {e}$ neurons to be excitatory and $N_\text {i}$ neurons to be inhibitory $(N_\text {t}=N_\text {e}+N_\text {i})$.

When an inhibitory neuron i fires, the synaptic connections with all other excitatory type neurons j are less than zero $(J_\text {ij}<0)$ so that inhibitory neurons do indeed inhibit excitatory neurons from firing. We use four times as many excitatory neurons as inhibitory $(N_\text {e}=4N_\text {i})$ as typically reported in cortical tissue^45^.

To start an avalanche, we raise the potential $V_\text {i}$ of all $N_\text {t}$ neurons by the amount $V_\text {thresh} - \text {max}(V_\text {i})$ such that the “origin” neuron i which is closest to the firing threshold $V_\text {thresh}=1$ is caused to fire. When this neuron i fires, the simulation resets its potential to a resting value $V_\text {i} \rightarrow V_\text {i}-\Delta V_\text {i}$. In the subsequent time step (we have checked that delaying this does not change our results, see Supplemental Sec. D) the firing event from neuron i then contributes to all other $N_\text {t}-1$ neurons the potential $\Delta V_\text {i}/(N-1)$.

The new failure threshold for an already fired neuron is weakened to a dynamical value $V_\text {dynamical} = V_\text {thresh} - \epsilon _{E/I} \Delta V_\text {i}$, with $0 \le \epsilon _{E/I} \le 1$. Note that this parameter is not necessarily the same for excitatory neurons as it is for inhibitory ones. Thresholds for the firing of a neuron during an avalanche are diminished due to this weakening process but are reset to their original default after an avalanche terminates. Thus, the model assumption relies on a separation of time scales between intra-avalanche and inter-avalanche firing (verified in the data, see Supplemental Sec. C) and assumes that some unspecified relaxation process allows for the resetting of firing thresholds on sufficiently long timescales after an avalanche terminates. As discussed below the separation of time scales does not have to be perfectly fulfilled to observe the predicted trends.

Avalanches emerge in this model when the origin firing event triggers one or more firing events in a cascading fashion. We identify the avalanche size *S* to be the number of total firing events, and *N* as the total number of neurons involved. All simulations were performed for 200,000 time steps each. To match both mouse and rat data sets we simulated system sizes from  450 to  2000 neurons and disorder strength $w=1.9$.

### Sub-sampling

In the experiment, the cortical slices are laid atop a 1 by 2 mm rectangular electrode array. Because only roughly one quarter of the total slice is being recorded by the MEA, there is chance that neurons outside of the field of view affect the recorded neurons i.e., by causing one of them to fire. To emulate this in our simulations, we have up-sized our system by a factor of four and then analyzed only one fourth of the neurons when constructing avalanches. We outline the sub-sampling procedure in more detail in Sec. E of the SI. We find that sub-sampling the data has the effect of increasing both the level of recurrent activity as well as the robustness of the clustered bi-test signature for small avalanches. In the SI, Fig. S9, we show that the sub-sampling alone cannot reproduce the data, and that weakening is indeed necessary for our simulations to reproduce the data.

## Results

### S vs N: evidence of recurrent activity

**Figure 2:**
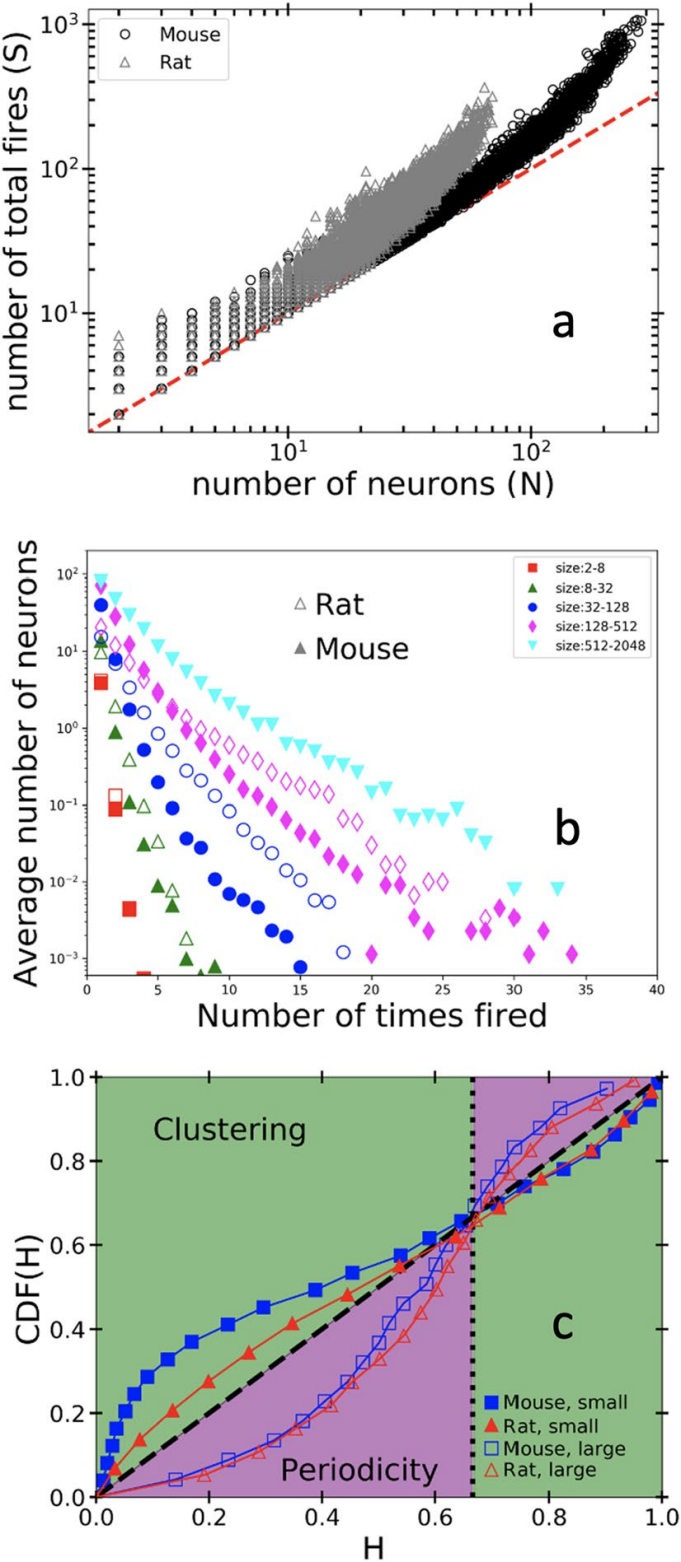
(**a**) Scatter-plot of avalanche size *S* as a function of the number of individual neurons *N* participating in each avalanche, as estimated by the causal-webs method^24^. For the mouse sample 434 neurons participated in 86,936 avalanches. For rat 106 neurons participated in 77,648 avalanches. (**b**) Distribution of the mean number of times each neuron fires in an avalanche, for avalanches within specified size bins. Note the rat data had no avalanches in the largest (size = 512–2048) bin. (**c**) Avalanche interevent time correlations. The cumulative distribution function of $H_\text {i}=\Delta t_\text {i}/(\Delta t_\text {i}+\Delta \tau _\text {i}/2)$, where $\Delta t_\text {i}$ is taken as the minimal interevent time, $\text {min}(|t_\text {i}-t_\text {i-1}|,| t_\text {i+1}-t_\text {i}|)$, and $\Delta \tau _\text {i}$ is the subsequent interevent time $( |t_\text {i-1}-t_\text {i-2}|$ or $|t_\text {i+2}-t_\text {i+1}| )$. Poisson distributed random interevent times fall directly on the dashed line, while perfectly periodic interevent times fall on the dotted line. The bi-test in the green regime is a signature of temporal clustering while in the purple regime is a signature of temporal quasi-periodicity.

Having reconstructed avalanches, we examine the relationship between the overall size *S* of an avalanche (total number of firing events) and the number of unique neurons *N* firing during that avalanche. For both mouse and rat samples, Fig. 2a, shows the relationship between the size *S* and number *N* participating in each avalanche. Smaller avalanches tend to involve approximately only a single firing event per neuron during an avalanche $(S \approx N)$, whereas the largest avalanches in this recording involve some neurons firing more than once $(S \gg N)$. Note that the mouse sample (black) shows more recurrent activity than the rat sample (*S*/*N* greater for the largest avalanches). Supplemental material shows similar plots for other samples and recordings (Fig. S2).

### Distribution of recurrent activity

We examine further the nature of these repeated firing events by considering the distribution of the number of times each neuron fires during an avalanche. Representative data are shown in Fig. 2b, for the rat and mouse samples, where we bin avalanches by their total size *S* and compute the mean number of neurons *F*(*m*) that fire m times during an avalanche. For any given avalanche bin (range of avalanche sizes), *F*(*m*) falls off roughly exponentially, but for larger avalanches, this exponential decay becomes slower and slower, with some neurons firing dozens of times in a single avalanche. In some samples (see Supplemental Fig. S3), some neurons fire hundreds of times during an avalanche.

### Cycles: the nature of recurrent dynamics

While the distributions shown in Fig. 2b demonstrate that some neurons fire many times during an avalanche, they do not reveal the structure of these avalanches or the role played by neurons firing many times. Are large avalanches characterized by many spontaneous (untriggered) firing events that propagate through the system in a feedforward manner, or are there cycles of feedback in which activity loops back to trigger previously active neurons? To address this question, we have probed the net- work structure of these neuronal avalanches to identify cycles of activity. Each avalanche consists of a directed graph whose nodes are particular neurons firing at particular times (i.e., (neuron, time) pairs), and whose edges connect nodes if there is an inferred causal link from a source node to a destination node (i.e., source contributes to the firing of destination). A time trace of one such large avalanche with recurrent activity is shown in Fig. 1. For our purposes here, a cycle is any directed path in an avalanche on which a given neuron fires at some initial time and then fires again at one or more subsequent times on the same directed path, implying a causal path connecting a neuron to itself at a later time.

In general, we find a complex, interwoven set of cycles, with multiple paths of activity leading into and out of recurrent loops, shorter cycles sometimes embedded in longer cycles, and injections of new activity from spontaneous activation events with no apparent triggering event. Characterizing the detailed structure of these cyclic patterns of activity is beyond the scope of the current work, but we can summarize the statistics of cycles as shown in Fig. 4, for both the mouse (a) and rat (b) samples examined previously. In particular, for any avalanche, we have calculated what fraction of (neuron, time) nodes in a causal web lie on at least one cycle, as defined above. Most small avalanches are completely feedforward with no cycles (the lower branch for small S), although some of the small avalanches do involve small cycles among a few neurons (the upper branch). As avalanches grow larger, they tend to have a larger fraction of firing events occurring within these cycles, and the mouse sample reveals a greater fraction of activity taking place within recur- rent cycles for the largest avalanches. Interestingly, recent work examined the same data set^46^, and although defining “cycles” in a different manner (more akin to our definition of “recurrence”), showed in simulations of networks that cascade duration increased with the number of embedded cycles, thus preserving information longer. In our analysis, working back from the data, we see that larger avalanches are associated not just with a greater amount of recurrent activity, but also a greater amount of cyclical/feedback activity, as we have defined it.

### Evidence of weakening

The Bi test extracts interevent time correlations that are used to investigate the presence of dynamical weakening. Here we use the Bi-test to show that the addition of dynamical weakening to our model produces a specific temporal signature: quasi-periodic interevent times between the large avalanches in the tail of the distribution Fig. 2c (see^42^). We note that a further signature is predicted; clustered interevent times between the small avalanches in the scaling regime, for our bare MFT model. We find, however, that using both inhibitory neurons as well as increased disorder strength causes this temporal signature to vanish. We outline in section F of the SI when the clustered signature does and does not show up in our model simulations (Fig. S10) and show how sub-sampling in the data could be a reason that we see bi-test signatures in *both* small and large avalanches in the data (See Fig. S11 and Sec. E in the SI). See Fig. S5 for results of the Bi-test on other samples.

### Model fit to *S* vs N

Adding dynamic weakening to the model enhances the possibility of recurrent activity, by successively lowering thresholds to activation and facilitating neuronal firing. We find this activity in both mouse and rat cortex, which manifests as an upward bending of the *S* vs. *N* curve away from the purely feedforward line (Fig. 2a). Model simulations without weakening produce *S* vs. *N* curves that follow this feedforward line (see Fig. S14, bottom, blue).Those with weakening however produce curves bent above the feedforward line (Fig. S14, bottom, red). We allow the inhibitory and excitatory weakening values to differ and find the optimal parameters for mouse and rat to be ($\epsilon _\text {e}=0.5$, $\epsilon _\text {i}=0.8$) and ($\epsilon _\text {e}=0.6$, $\epsilon _\text {i}=0.7$), respectively. With these parameters alone, we find the *S* vs. *N* distributions bend, although there are larger avalanches in the experiment, and thus the tails of the scatter plot do not match (See Fig. S14, top, red).

**Figure 3:**
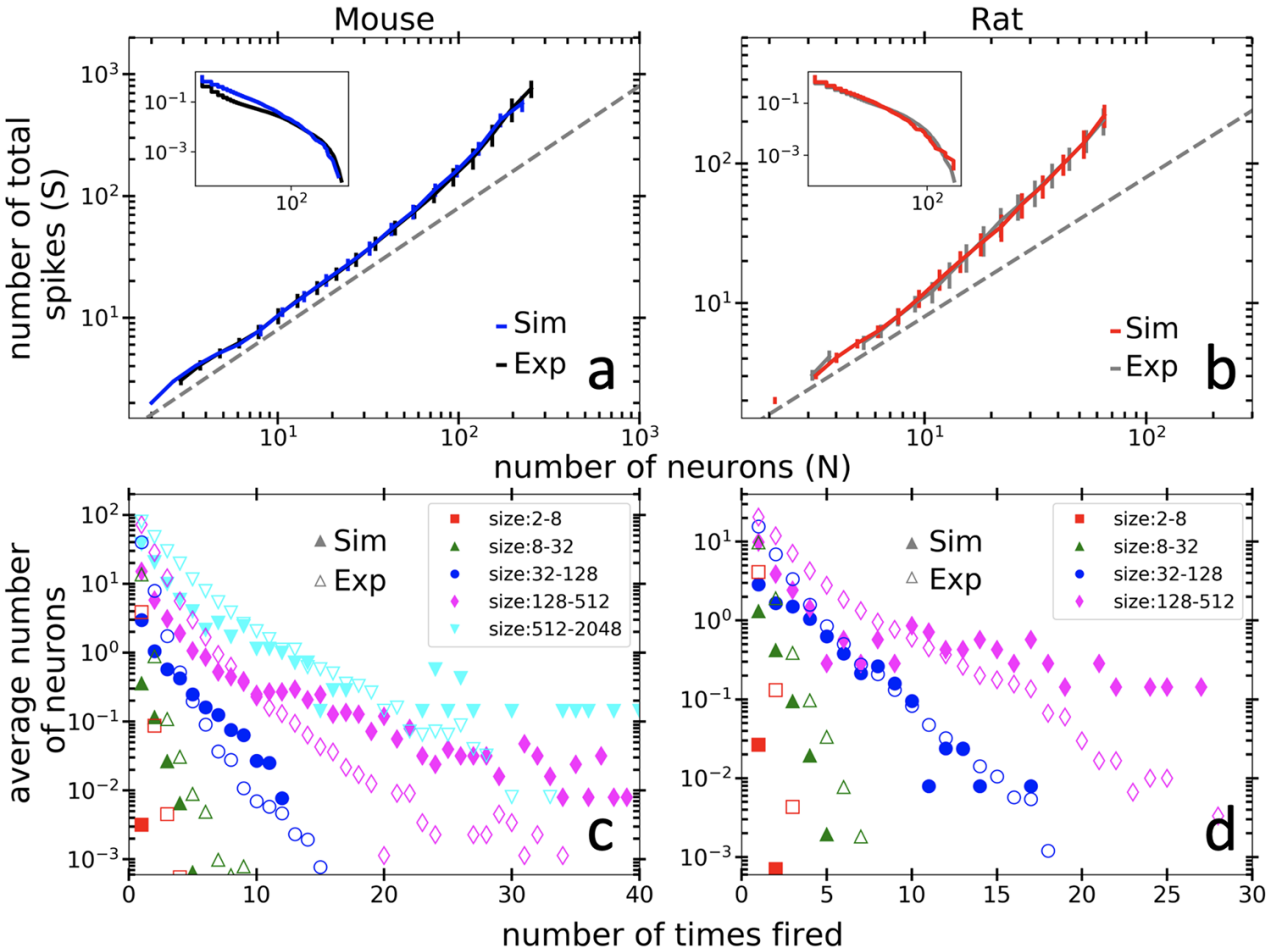
Recurrent activity in mouse cortical neurons. Causal web analysis of neuronal avalanches in both mouse (black line, **a**) and rat (grey line, **b**) are shown in comparison to model simulations (blue and red lines, respectively). Lines represent the average value of size, *S*, within bins of the number of neurons, *N*. We use twenty logarithmically spaced bins and error bars show the standard deviation of the bin. Using $\epsilon _\text {e}=0.5$ $\epsilon _\text {i}=0.8$ the model captures the recurrent activity and matches the size vs. *N* distribution in the mouse. Adjusting the number of neurons in the simulation and the weakening parameters ($\epsilon _\text {e}=0.6$ $\epsilon _\text {i}=0.7$) the model fits the rat distribution as well. We reduced the inhibitory-excitatory coupling strengths by a factor of $\gamma =4.8$ and $\gamma =2.5$ for the mouse and rat simulations, respectively. The complement of the cumulative distribution for avalanches size *S* (C(S)) is shown in the inset compared to the simulated distribution, which qualitatively (power law) and quantitatively (− 1 slope) match the experiments, for mouse and rat both. Note that the avalanche sizes involved in the mouse experiments were larger than those in the rat, so the axes in the right span a smaller range for qualitative comparison of the distributions in different species. Mouse (**c**) and rat (**d**) neurons fire multiple times in an avalanche. The model simulations (empty markers) qualitatively follow the broadening of the experimental distributions (filled markers) signifying recurrent activity. Importantly, the simulations also broaden more for larger size avalanches, which is seen in the experiments facilitates the bending in Fig. 1,a. No avalanches existed in the largest size bin (128–512) for the rat, and the axes are different between left and right for better qualitative comparison. All simulations were run for 200,000 time steps with a disorder strength, $w=1.9$.

By reducing the coupling strength between excitatory and inhibitory neurons by a factor of $\gamma$ (Fig. S14, top, green), we effectively enhanced the network excitability, leading to larger avalanches for a better fit to the tail of the experimental scatter plot Fig. 3a and b. For the mouse and rat data, we found good agreement with $\gamma =4.8$ and $\gamma =2.5$, respectively.

### Model fit to firing distribution

With the addition of dynamic weakening, the model is able to qualitatively capture the phenomena of multiple neuronal firing. To further investigate the structure of recurrent firing we have constructed the distribution of single neuron firing times for every avalanche which we then averaged together using bins in size, analogous to the data presented in Fig. 2b. As with the experimental data, we see that larger avalanches in the model are associated with a widening of the distribution of neural firing (i.e., neurons are more likely to fire repeatedly). With these parameters, however, the simulations somewhat overestimate the probabilities in the tails of the distributions (Fig. 3c,d). We note that this widening of the distribution of neural firing in our model is very slight unless we add weakening (see SI, Fig. S15).

### Recurrent cycles in model simulations

**Figure 4:**
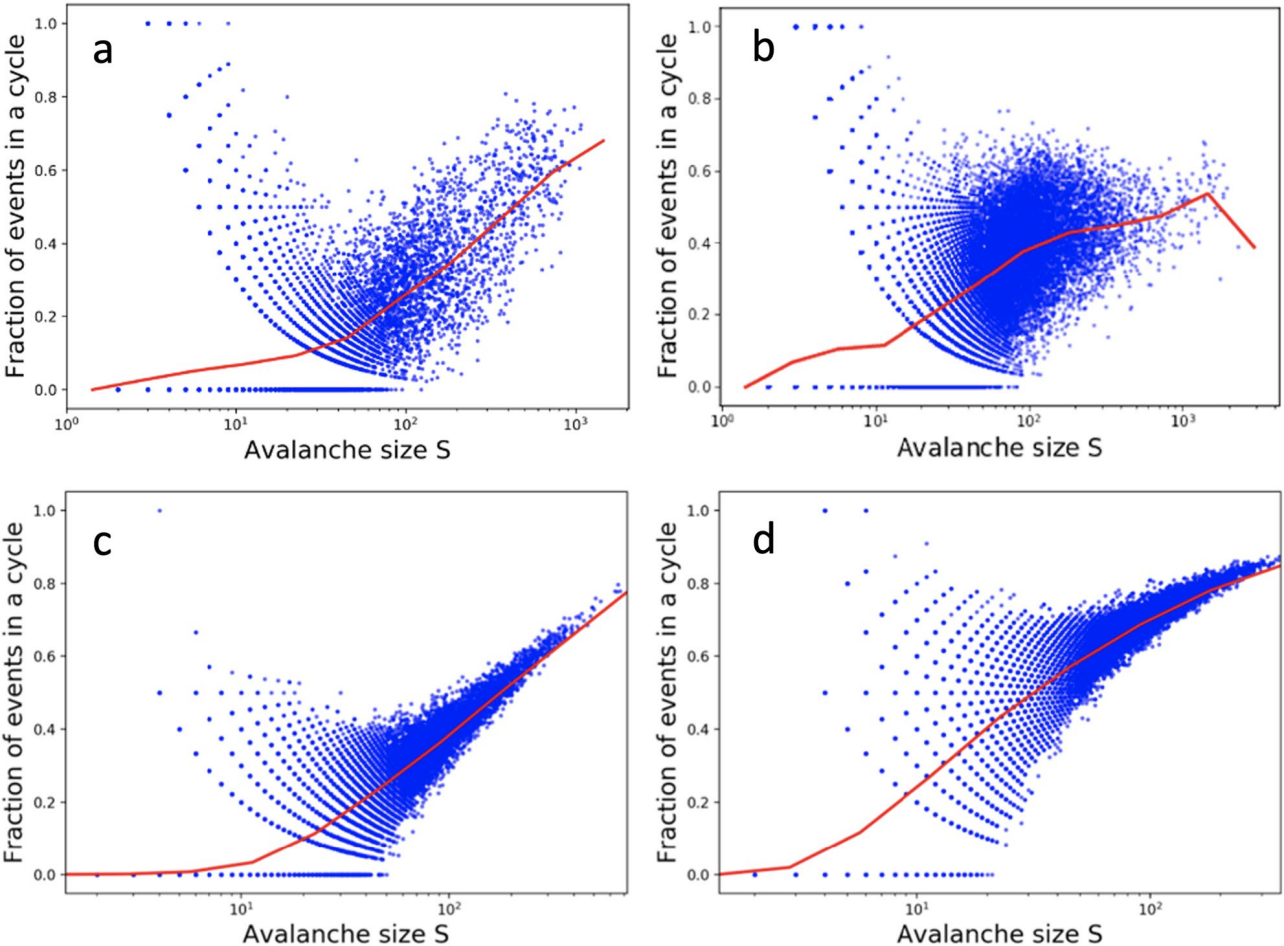
Fraction of firing events that lie on a cycle of activity, as a function of overall avalanche size *S*, for mouse experiments (**a**), rat experiments (**b**), mouse simulations (**c**) and rat simulations (**d**). By detecting cyclical activity of firing events on directed causal paths (see text), all firing events are classified as either lying on a cycle or not. For each model simulated avalanche, the fraction of firing events in a cycle for that avalanche is plotted as a function of avalanche size. Large avalanches are characterized by more recurrent firing, and a greater fraction of firing lying on cycles. Blue dots represent individual firing events within an avalanche, and the red lines represent the mean fraction of events in a cycle for all avalanches within a corresponding size bin.

We analyzed the dynamical trajectories generated by the simulation model to assess the presence of cycles, as for the experimental data. In the model, a given set of neurons fire at one discrete time step, leading to the firing of a different set of neurons firing at the next time step. Therefore the “causal webs” that we infer from the model trajectories are more densely connected than those we infer from the experimental data, since each neuron firing at one time step contributes in principle to the activity of all firing neurons at the next time step. Nonetheless, we have computed the fraction of model firing events that lie on a cycle as a function of avalanche size, and plotted those results in Fig. 4c and d for mouse and rat parameters. These results on top (experiments) are qualitatively similar to the analogous plots on bottom (model simulations), revealing the merging of distinct feed-forward and recurrent branches of activity for small avalanches, and an increase in cyclic activity as avalanches grow larger.

## Discussion

Here we have demonstrated that dynamic threshold weakening can induce recurrent firing patterns in cortical neurons. This dynamic reduction of firing thresholds is reminiscent of structural weakening in materials models^14^ and has been inferred from parameter estimation algorithms applied to current clamp recordings of neurons^13^. Importantly we note that in addition to dynamic threshold weakening, there is also evidence for a firing frequency adaptation mechanism^13^. Since recurrent activity emerges at the onset of a seizure^20^ we could use this effect, whatever its origins, to understand and combat dangerous pathologies such as epileptic activity.

These basic patterns were observed in multiple tissue samples, from both rat and mouse. The similarity of these two example cases suggests much broader applicability of the results. For both mouse and rat data, the same model qualitatively reproduces the bending of the *S* vs *N* curves (Fig. 3a,b), the broadening of the distribution of multiple neuron firing events (Fig. 3c,d) and the propagation of activity around cycles (Fig. 4c,d). Note that the relationship between avalanche size and the time interval between avalanches (previously studied in^2^), is separate from the relationship between avalanche size and the intervals of recurrent firing events within them (here called cycles). With very minimal tuning of the weakening parameter between species, the model comparison can even be quantitative (e.g., it predicts the maximum values of avalanche sizes for a given network size, maximum number of times one neuron will fire in a given avalanche, etc.). This robust confirmation across data sets indicates that this weakening phenomenon is likely a general feature of neuronal avalanches in cortex.

Interestingly, other systems in nature also display weakening effects, which might help provide intuition about the dynamics of neuronal systems. Weakening has been observed in a wide variety of systems, and it is often associated with dynamical instabilities, spatio-temporal pattern formation, and spatial localization. Velocity-weakening friction in models of earthquakes leads to localized pulses of slip that propagate along faults^27^. Shear localization in materials involves spatially concentrated bands of shear deformations triggered by weakening^28^. Formation of river basins proceeds through a feedback process of canalization whereby locally increased water flow results in increased soil erosion, thereby supporting greater flow through emergent channels^29,30^. We therefore speculate that weakening in neuronal networks might show similar dynamics, with channels of recurrent neural activity carved out by the avalanche dynamics itself, a scenario that can be studied in future work.

To strengthen the model, different biologically inspired amendments could be considered. For instance, the topology of neuronal systems is not all-to-all as we have assumed in this work for simplicity. Whether large avalanches and their recurrent activity are affected by topology in the model presented here could be the subject of some interesting future work. Further, our instant re-healing of a firing threshold after an avalanche terminates was ultimately used to limit the timescales introduced in the problem, but could instead in the future be modeled by a slower re-healing process. We lastly point out that results in-vivo, where the separation of timescales assumed here is not likely to be true, have never the less been captured by models that require such a separation in timescales^4,11,12,33^, supporting the claim that our results may too be applicable in-vivo.

## Conclusion

Here we have shown that the larger avalanches in neuronal networks involve distributions of recurrent electrical activity, with multiple feedback cycles permeating the avalanche dynamics. We have demonstrated that a dynamic weakening of thresholds for neuronal activation is a plausible explanation for the experimental neuronal firing data.

Our model was purposefully designed as simply as possible to reproduce the trends in the data. That way we could extract the key components of the underlying mechanism for the recurrent behavior, and make predictions for universal aspects of the dynamics, that do not depend on the microscopic details. Future work could include the microscopic details of specific neuronal systems and refine the model for detail dependent predictions.

Our simple coarse-grained model with weakening reproduces patterns of recurrent activity observed in the data without the need to include intrinsic neural dynamics. A Bi-test applied to the data, and the fits of the avalanche model with weakening, both support the notion of a dynamical weakening mechanism. This weakening mechanism leads to the recurrent operation of neurons and the formation of large “runaway” neuronal avalanches. For large weakening these runaway avalanches sweep most of the neurons – reminiscent of pathologies such as epileptic seizures.

## Supplementary Information

Supplementary Information.

## Data Availability

The datasets analysed during the current study are available in the CRCNS—Collaborative Research in Computational Neuroscience—Data sharing repository, accession: ’ssc-3’, link: http://crcns.org/data-sets/ssc/ssc-3/about-ssc-3.
