# Supplementary material for: Recurrent activity in neuronal avalanches
Source: Sci Rep. 2023 Mar 24;13:4871. doi: 10.1038/s41598-023-31851-x (PMC10039060; doi:10.1038/s41598-023-31851-x)
Supplement: Supplementary file 1 — Supplementary Information. [file 41598_2023_31851_MOESM1_ESM.pdf]

Supplemental Material for "Recurrent activity in neuronal avalanches"

Tyler Salners, Karina E. Avila, Benjamin Nicholson, Christopher R. Myers, John Beggs, Karin A. Dahmen

February 10, 2023

A: Filtering for criticality and experimental robustness

The experiments that show agreement with the predicted -1 power-law decay of avalanche size CCDF were used as critical data sets. Figure 1 shows the critical data sets not used in the main paper. S vs. N curves for all critical mouse and rat data show the characteristic bending (Figure 2). Firing time distributions for all experiments show multiple firings (recurrent activity) in both rat and mouse (Figure 3). Cycle (Figure 4) and Bi-test (Figure 5) plots looks the same for all experiments.

B: Artifacts of fire signal processing

Activation of a neuron in separate avalanches can be falsely identified as recurrent activation if caution is not taken during fire signal postprocessing. Traditionally, fire signals of continuous time are coarse grained into bins of width δt, after which an avalanche is defined as a set of consecutive nonempty bins (bounded by empty bins) [1]. With this definition, two simultaneous yet separate avalanches involving neuron i can be processed into a single merged avalanche. Therefore, the recurrent activation (of neuron i) is only an artifact of this merging. Causal webs processing avoids this ambiguity [6] whereby only causally connected strings of fires are inferred to be avalanches. Accordingly, for a fire train analyzed via the traditional time binning method, we would expect to see more recurrent behavior as compared to the same signal analyzed via causal webs. Using a time bin width of 5 milliseconds we confirmed this hypothesis in the mouse data set (Figure 6). Thus, this method helps to assure that we are reporting something physical and not just an artifact of post processing.

C: Inter and Intra-avalanche firing timescales

Our model assumes that there is sufficient time between avalanches to allow for firing thresholds to be reset to their initial value after an avalanche ends, implying that typical intra-avalanche firing times are more tightly clustered than

are inter-avalanche firing times. Here we investigate that assumption. From the mouse data set we extracted the inter-fire times of each neuron and calculated both the average time between fires within the same avalanche and the average time between fires that were not in the same avalanche. In Figure 7 We plot the mean of each neuron below, with error bars showing the standard error of the mean. For each neuron, the average time between intra-avalanche fires (red) is orders of magnitudes smaller than the average time between inter-avalanche fires (green). This separation of the means was verified for each neuron with a hypothesis test that the two means were the drawn from the same normal distribution. We found the hypothesis to be rejected ($p \sim 0$) for all neurons in the system. Thus, the assumption of these separated timescales has been justified for our data. We note that causal webs' do allow for simultaneous avalanches– yielding the possibility of a neuron having negative inter-avalanche firing times. We show in magenta the averages with these negatives values removed and see that the change to the results are negligible.

D: Synaptic time delay in simulations

Our model assumes that activity happening during an avalanche is much quicker than the time between consecutive avalanches i.e., that the neurons effectively communicate instantaneously. Here we use the model simulations to understand how our results change with the addition of a more biologically accurate synaptic time delay. In the original simulation protocol, the ith neuron firing at time t affected all other neurons j at time t+1. We show in Figure 8 the effect of delaying this until time $t + \tau$ for $\tau = 1, 2, 4, 20$ simulation steps. Though we find that increasing time delay decreases the avalanche size cutoff, the effect of recurrent activations persists as an upward bending of the S vs. N curve.

E: Sub-sampling

The experimental setup suggests that roughly one quarter of the total number of neurons in the cortex slice are being recorded by the MEA. This is not because the electrode density is too sparse to see all of the neurons; in fact the electrode density is so high, all of the neurons above it can be identified with very high accuracy through triangulation [3]. The other three quarters of the neurons actually come from the other three quarters of the cortex slice that is *not* directly above the MEA. So, we have neurons outside of the view ('outside' neurons) and neurons inside the view ('recorded' neurons). Not recording the entire cortical slice allows the possibility of an 'outside' neuron firing and causing a 'recorded' neuron to fire as well.

To account for this in our model, we performed a simple sub-sampling procedure on our simulation output that allowed us to emulate this experimental detail; namely that the experiment involved an entire slice but only recorded from a quarter of it. If our experimental system consisted of N 'recorded' neurons, we would simply simulate $4N$ neurons, but only consider firing events from every fourth neuron in the system. This way, when we construct the avalanches,

neurons are truly receiving information from 'outside' neurons, which causes them to fire seemingly unprovoked. We have thus emulated the experimental situation of neurons from outside the field of view influencing neurons within the field of view, some times referred to as a 'sub-sampling' effect.

The results in the main paper (Figure 3 a,b) are shown for simulations that had this sub-sampling procedure performed and we can see again consistency between the data and experiment. We have also verified that this sub-sampling was not affecting the presence of the recurrent activity. We show in Figure 9,a how the S vs. N curves change as we sub-sample. We find that the sub-sampling procedure enhances the recurrent activity in our simulations. We point out however that this enhancement through sub-sampling is not sufficient to account for the extent of the recurrent activity seen in the experiments *alone* (Figure 9,b). We can reproduce experimental avalanche size CCDF's and S vs. N curves with our simulations with weakening alone or with weakening including sub-sampling, but not with sub-sampling alone. We also find the sub-sampling procedure can induce a clustered bi-test signature in simulations where it once was not present without sub-sampling, this trend is described in detail in the next section.

F: Bi-test for model simulations

Our simple slip model with weakening has been shown through large system-size simulations to admit two unique signatures related to temporal correlations between avalanches [see chapter 2 in [5]]. Specifically, predictions about the intervals between consecutive avalanche start times can be made which admit a signature that differs between small and large avalanches. This signature, the bi-test of inter-avalanche start times (outlined in the manuscript), for small avalanches is either Poissonian when there is no weakening (Figure 10, Blue), or temporally clustered if there is weakening (Figure 10, Red). Though these signatures are seen in the bare MFT model, we can make the clustering vanish by making the width of the disorder distribution large enough (Figure 10, Green). Further, once we add inhibitory neurons, the clustering signature is almost certain to vanish (Figure 10, magenta). With both inhibitory neurons and high disorder width, as we need for our simulation to match the model, we also fail to recover clustered small avalanche inter-start times (Figure 10, cyan).

Since the clustered signature seen in the data is not predicted by our simulations (i.e., to be a result of weakening), what causes it? We have investigated the effects of sub-sampling the simulations to more closely replicate the experiments (see Section E above). We find that, under sub-sampling of our simulations, the clustered bi-test signature for small avalanches is recovered (Figure 11). We were not able to get a sub-sampled simulation to all at once match the size distribution, S vs. N and the Bi-test, but we propose that sub-sampling is a possible reason for the appearance of the clustering signature in the data. We leave a perfect implementation of a sub-sampling scheme to future work, for this paper, we simply show that it is a possible explanation.

G: Disorder Configurations

Each time we run the simulation, a new set of arrest stresses is drawn from a uniform distribution. This means that each simulation will not have exactly the same evolution over time. Specifically, the stress configuration of the system can stay relatively uniform for the duration of the simulation, or it can evolve into a bunched state; see [2]. This can result in a handful of large events (significantly larger than the rest) obscuring the fit to experiment (Figure 12, disorder realization $\#'s$ 1,3-6,8-10). Importantly the statistical features we are concerned with, for instance, the power-law slope of the avalanche size CCDF, do not change with these different realizations of disorder (Figure 12). With that being said, the experiments have one realization of disorder, which we are apriori unaware of. For this reason, in the main paper (Figure. 4), we highlight one disorder configuration that compares best with the experiment (disorder realization #2, Figure 12).

References

[1] John M. Beggs and Dietmar Plenz. "Neuronal Avalanches in Neocortical Circuits". In: *The Journal of Neuroscience* 23.35 (Dec. 2003), pp. 11167–11177. ISSN: 0270-6474. DOI: 10.1523/JNEUROSCI.23-35-11167.2003. URL: http://www.jneurosci.org/lookup/doi/10.1523/JNEUROSCI.23-35-11167.2003.

[2] Daniel S. Fisher et al. "Statistics of Earthquakes in Simple Models of Heterogeneous Faults". In: *Physical Review Letters* (1997). ISSN: 10797114. DOI: 10.1103/PhysRevLett.78.4885. eprint: 9703029 (cond-mat).

[3] Shinya Ito et al. "Large-scale, high-resolution multielectrode-array recording depicts functional network differences of cortical and hippocampal cultures". In: *PLoS ONE* 9.8 (2014). ISSN: 19326203. DOI: 10.1371/journal.pone.0105324.

[4] Louis W. McFaul et al. "Applied-force oscillations in avalanche dynamics". In: *Physical Review E* 101.5 (2020), pp. 1–10. ISSN: 24700053. DOI: 10.1103/PhysRevE.101.053003.

[5] William McFaul. "TEMPORAL PROPERTIES OF AVALANCHE DYNAMICS". PhD thesis. University of Illinois at Urbana Champaign, 2016.

[6] Rashid V. Williams-García, John M. Beggs, and Gerardo Ortiz. "Unveiling causal activity of complex networks". In: *EPL (Europhysics Letters)* (2017). ISSN: 0295-5075. DOI: 10.1209/0295-5075/119/18003. eprint: 1603.05659.

G: Figures

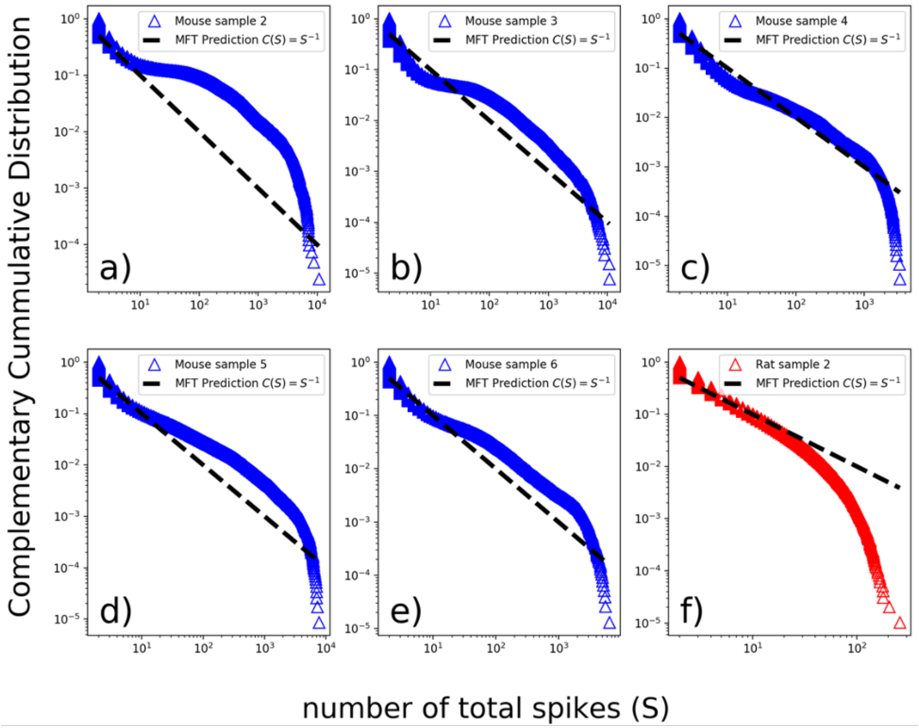

Fig. S 1: **Complementary cumulative distribution function (CCDF) of avalanche size S.** Fire signals of cultured cortex recorded with a 512-electrode array and processed into causal webs were used to extract avalanche sizes (S). The CCDF in size S (number of total fires) is shown here for five critical mouse sets (a-e) and one critical rat set (f) not shown in the main paper. The critical distributions follow a power law on a log-log scale with exponent minus one, shown here as a dashed line.

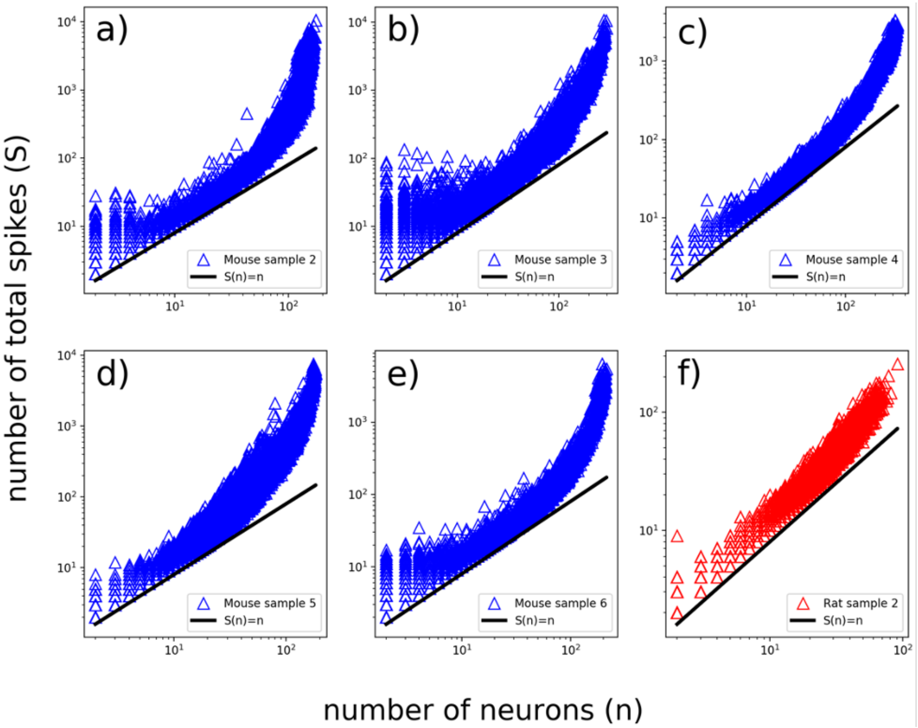

Fig. S 2: **Bending in S vs. n.** Fire signals of cultured cortex recorded with a 512-electrode array and processed into causal webs were used to extract from each avalanche the number of unique neurons (n) and size (S). The two are plotted vs. each other above on a log-log scale, with the black line S(n)=n, shown here for five critical mouse data sets (a-e) and one critical rat data set (f) not shown in the main paper.

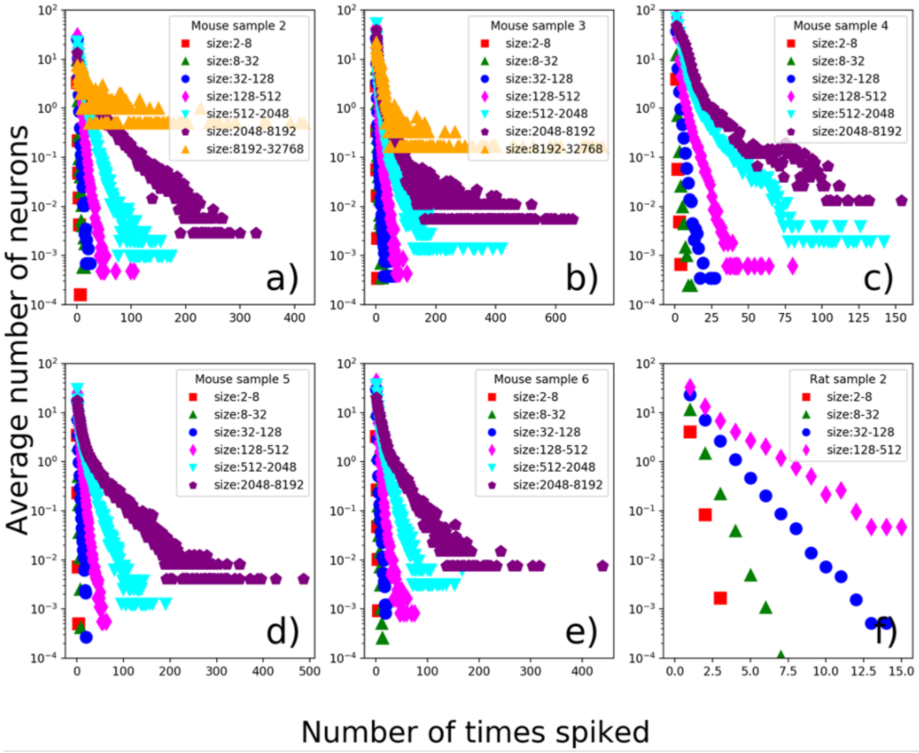

Fig. S 3: **Distribution of multiple firing events.** Fire signals of cultured cortex recorded with a 512-electrode array and processed into causal webs were used to extract the distribution of the mean number of times each neuron fires in an avalanche, for avalanches within specified size. They are shown here for five critical mouse data sets (a-e) and one critical rat data set (f) not shown in the main paper.

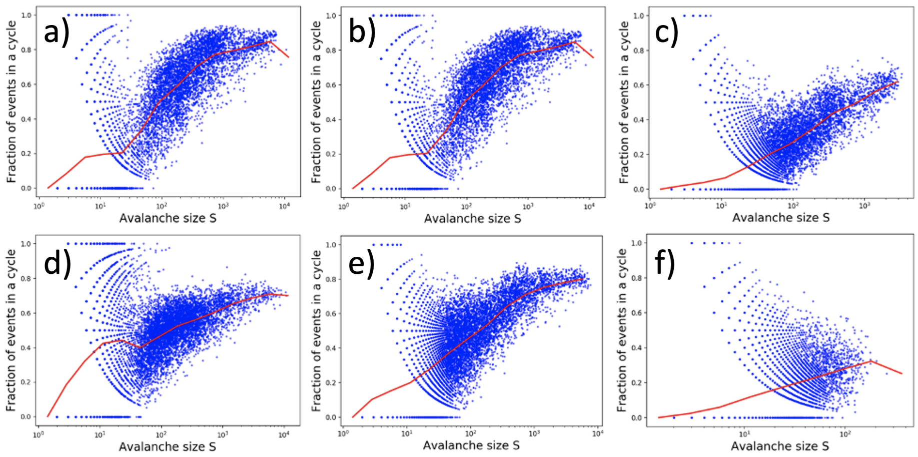

Fig. S 4: **Cycles in avalanches.** Fire signals of cultured cortex recorded with a 512-electrode array and processed into causal webs were used to extract cycles of recurrent activity shown here for five critical mouse data sets (a-e) and one critical rat data set (f) not shown in the main paper.

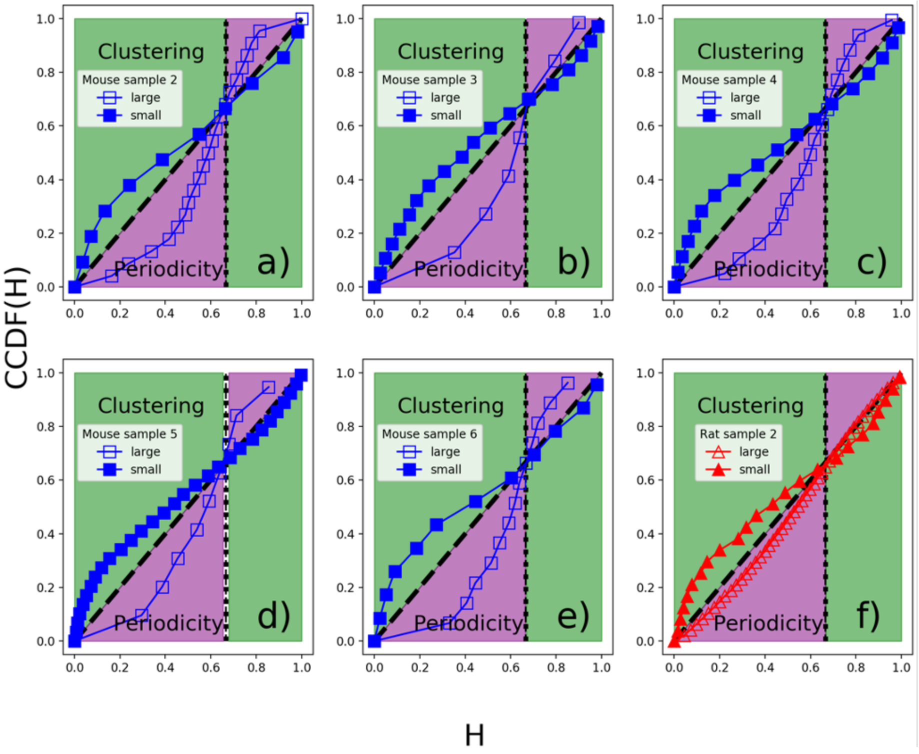

Fig. S 5: **Bi-test.** Fire signals of cultured cortex recorded with a 512-electrode array and processed into causal webs were used to extract avalanche interevent-times. The cumulative distribution function of $H_i = \Delta t_i/(\Delta t_i + \Delta\tau_i/2)$ is shown here for large (empty markers) and small (filled markers) avalanches. Δt_i is taken as the minimal interevent time, $\min(|t_i - t_{i-1}|, |t_{i+1} - t_i|)$, and τ_i is the subsequent interevent time ($|t_{i-1} - t_{i-2}|$ or $|t_{i+2} - t_{i+1}|$). Poisson distributed random interevent times fall directly on the dashed line, while perfectly periodic interevent times fall on the dotted line. The green regime is a signature of temporal clustering while in the purple regime is a signature of temporal quasi-periodicity. They are shown here for five critical mouse data sets (a-e, blue, square) and one critical rat data set (f, red, triangle) not shown in the main paper.

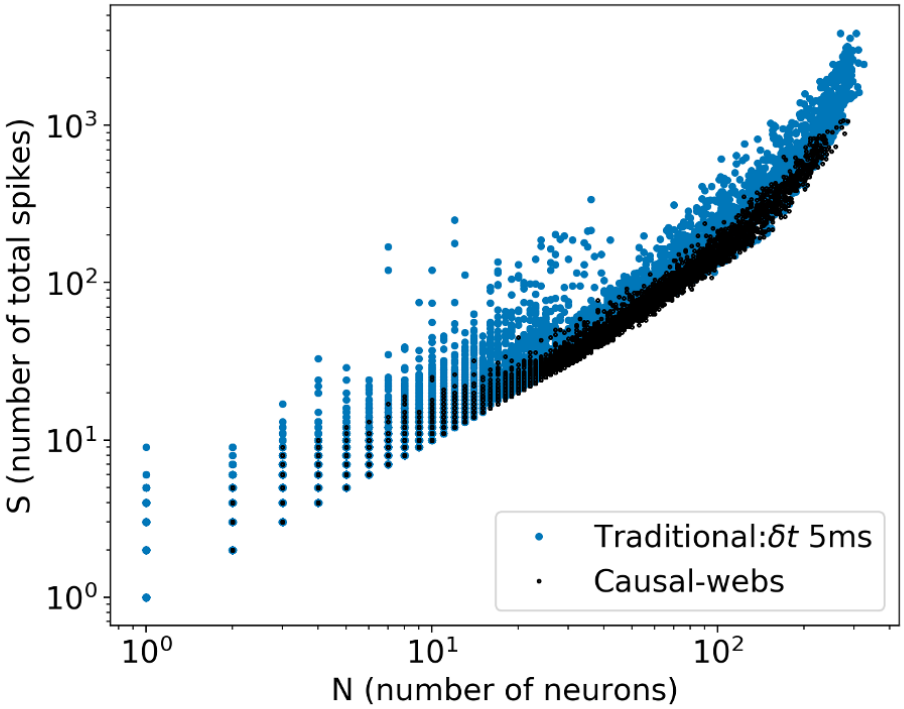

Fig. S 6: **Traditional avalanche definitions over-emphasize recurrent firing.** Fire signals of cultured mouse cortex recorded with a 512-electrode array processed two ways: (blue) sorting fires into time bins of width δt, defining an avalanche as any consecutive set of non-empty bins bounded by empty bins [1]. (black) sorting fires into causally connected space (neuron)-time pairs, defining an avalanche as any string of causally connected fires [6]. Avalanche sizes are plotted versus the number of unique neurons that fired in the avalanche.

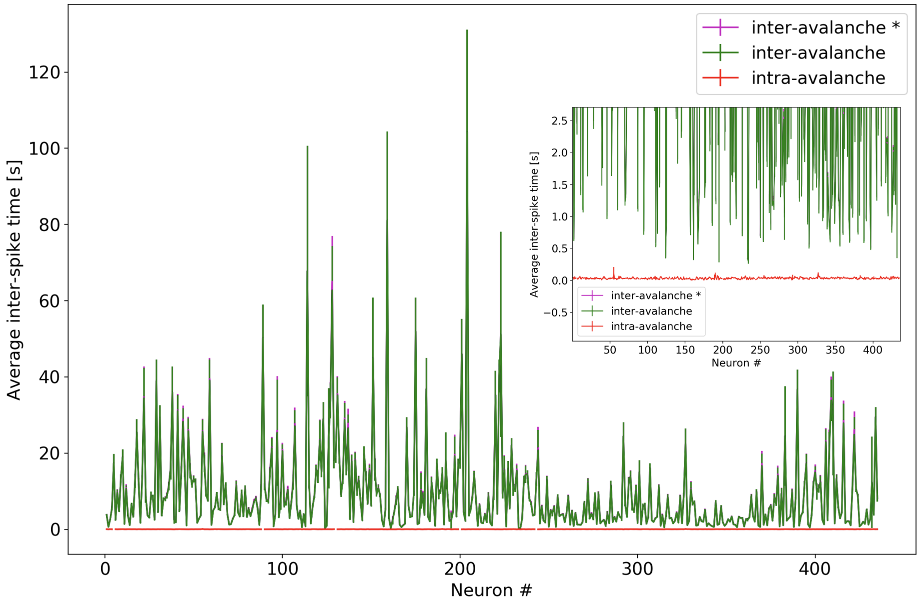

Fig. S 7: **Neurons fire more frequently within an avalanche.** Single neuron average inter-fire time within avalanches (intra, red) and between consecutive avalanches (inter, green) are plotted for all 434 neurons in the mouse cortex sample. Error bars show the standard error of the mean. The average with negative values removed is plotted in magenta and labelled as 'inter-avalanche*'. (inset) y-axis zoom in to show the separation of timescales.

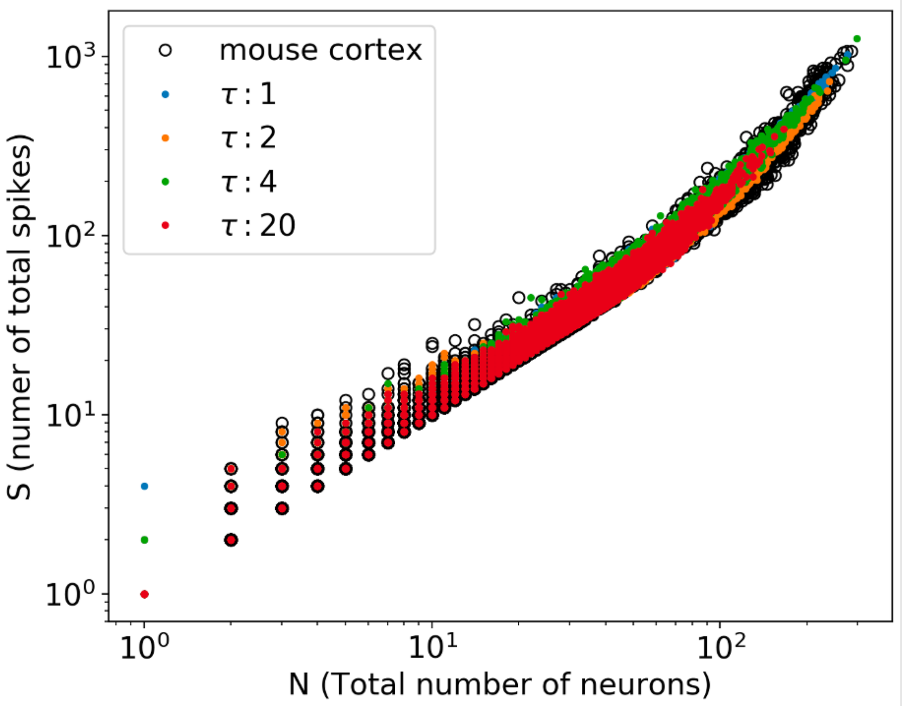

Fig. S 8: **Synaptic time delays do not hinder recurrent activity in model simulations.** The number of fires (S) is plotted versus total number of neurons (N) for different synaptic time delays $\tau = 1, 2, 4$ and 20.

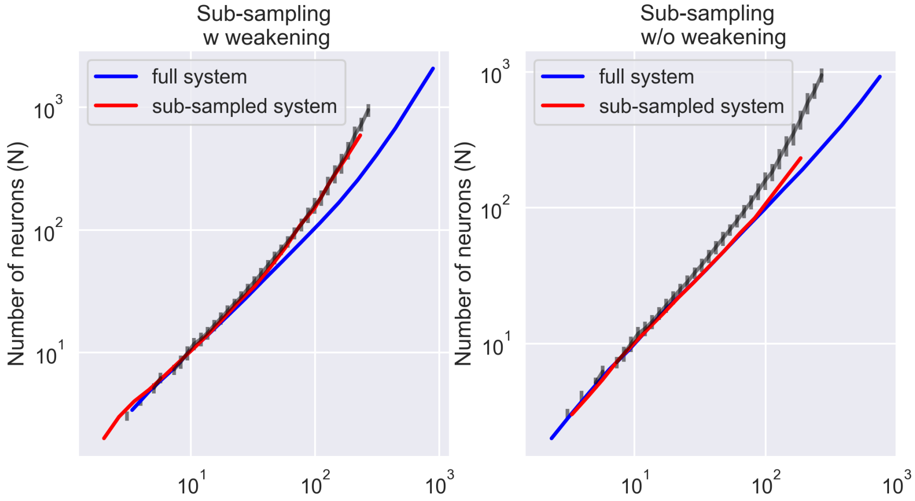

Fig. S 9: **Clustered Bi-test signature is restored by sub-sampling.** Simulations of the mouse system (N=434) were ran with W=1.9, $\epsilon_e = 0.5$ $\epsilon_i = 0.7$, $\gamma = 4.8$. (Left) simulations with nonzero weakening show the sub-sampled system has good agreement with experiment (red line). (Right) the sub-sampled system without weakening does not replicate the data nearly as well as the simulations with weakening (red line). From this figure we conclude that sub-sampling alone cannot account for the recurrent activity seen in the data.

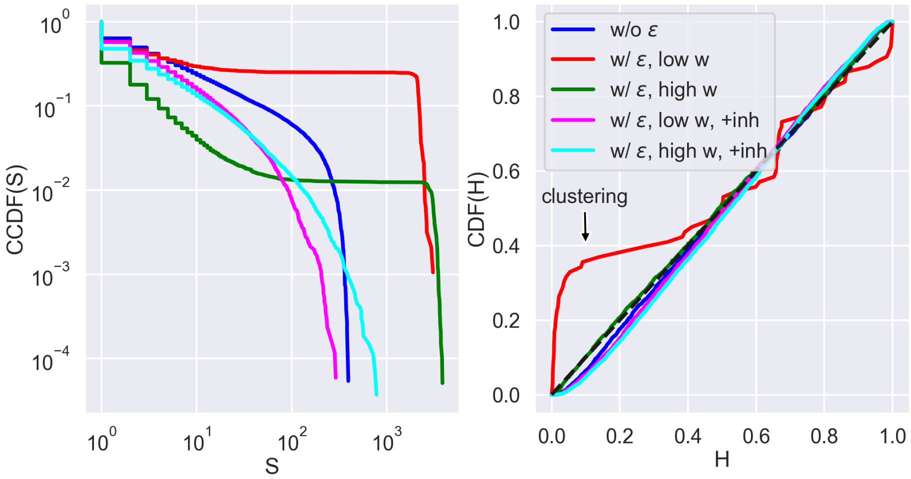

Fig. S 10: **Clustered Bi-test signature is destroyed by disorder and inhibition.** Simulations of the mouse system (N=434) were run with low disorder (W=0.1), $\epsilon_e = 0.5$ $\epsilon_i = 0.7$, $\gamma = 4.8$. With out any weakening, the small avalanches show a near Poissonian bi-test signature (blue). With the addition of weakening, the signature resembles clustering (red). However, the addition of strong disorder (W=1.9, green) or inhibitory neurons (magenta) or both (cyan) leads to the destruction of this clustered signature. From this investigation we provide a possible explanation for why the clustered signature is not present in our model simulations that include inhibitory neurons and large disorder.

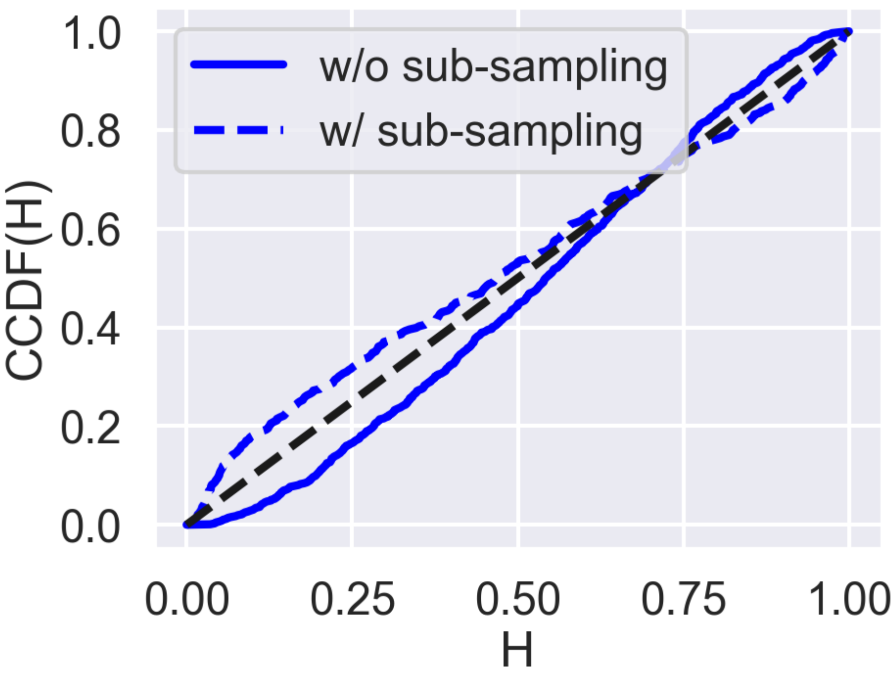

Fig. S 11: **Clustered Bi-test signature is restored by sub-sampling.** Simulations of the mouse system (N=434) were run with W=1.9, $\epsilon_e = 0.5$ $\epsilon_i = 0.7$, $\gamma = 4.8$. The solid line shows the full simulation (with $4N$ neurons) and the dashed line that same simulation after being sub-sampled by a factor of 4. From this figure we conclude that sub-sampling could be partially responsible for the clustered signature we see for small avalanches in the data.

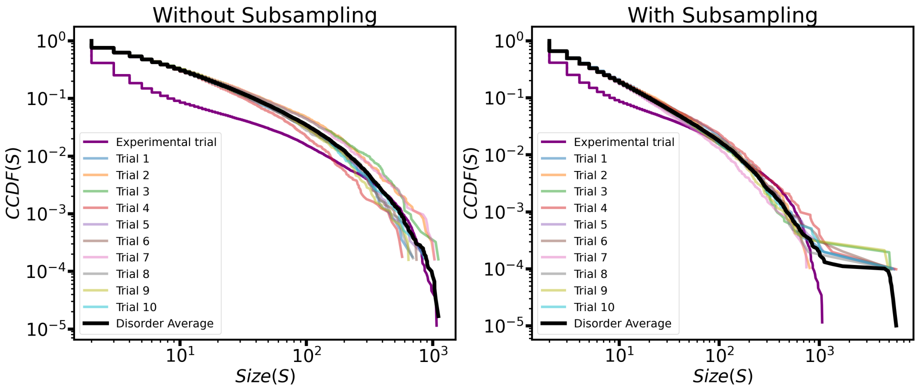

Fig. S 12: **Disorder Average.** We show the CCDF of avalanche size for 10 different realizations of disorder (or 'trials') in varying colors. The experimental trial from the main paper, mouse, is shown in purple and the disorder averaged CCDF is shown in black. The left figure shows the CCDF with out sub-sampling, the right figure shows the CCDF with sub-sampling. All configurations on the left match the tail of the distribution fairly well, along with the disorder average. There is discrepancy for smaller sized avalanches though, and this discrepancy seems to relax with sub-sampling (right figure). The trade off is that there are sometimes large avalanches in the sub-sampled (and accordingly quadrupled in size) system, as explained in the SI, Sec G. Still, an individual simulation trial (trial 2 and 7, for instance) compare quite well to one experimental trial.

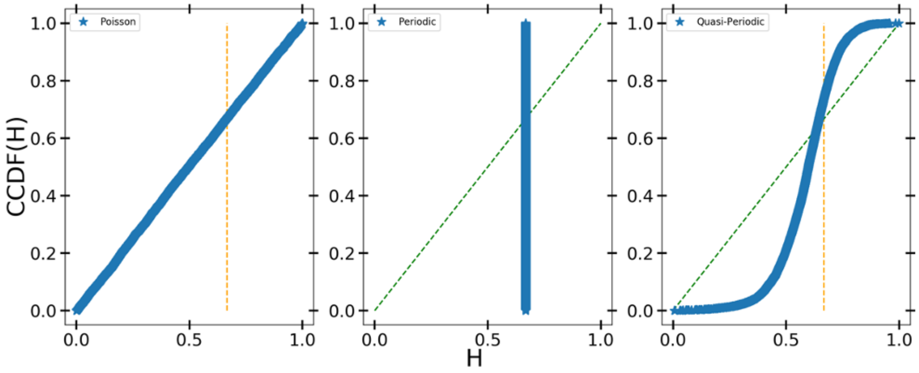

Fig. S 13: **Calibrate the Bi-test.** The cumulative distribution function of $H_i = \Delta t_i/(\Delta t_i + \Delta\tau_i/2)$, where $\Delta t_i$is taken as the minimal interevent time, $\min(|t_i - t_{i-1}|, |t_{i+1} - t_i|)$, and τ_i is the subsequent interevent time ($|t_{i-1} - t_{i-2}|$ or $|t_{i+2} - t_{i+1}|$) shown here for example Poissonian (left), periodic (center) and quasi-periodic (right) fire-trains. For applications of the Bi-test to plasticity see [4].

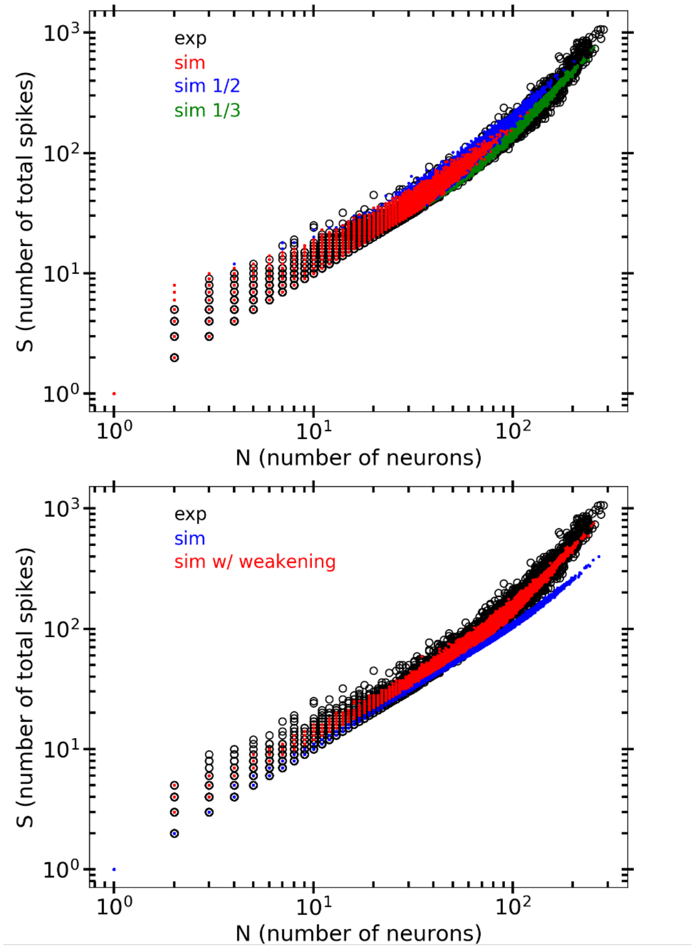

Fig. S 14: **Reducing the strength of inhibitory couplings and adding dynamic weakening.** Simulations of the mean field model were run with 108 inhibitory neurons and 326 excitatory neurons connected by a weight J_{ij} drawn from a log normal distribution $P(J_{ij}) = \frac{1}{J_{ij}\sqrt{2\pi N_t}} e^{\frac{-ln(J_{ij})^2}{2N_t}}$, where N_t is the total number of neurons and a factor of -1 is added when neuron i and j are not of the same type. Avalanche sizes are calculated, along with total number of participating neurons, shown here plotted against each other on a loglog scale. Fire signals of cultured cortex recorded with a 512-electrode array and processed into causal webs were used to extract from each avalanche the number of unique neurons (n) and size (S) shown in black circles. (Top) Non-symmetric coupling: The excitatory to inhibitory weights were reduced by a factor of one (red dots) one half (blue dots) and one third (green dots). (Bottom) Weakening needed for bending in model: The model simulations are plotted with (red dots) and without (blue dots) dynamical weakening.

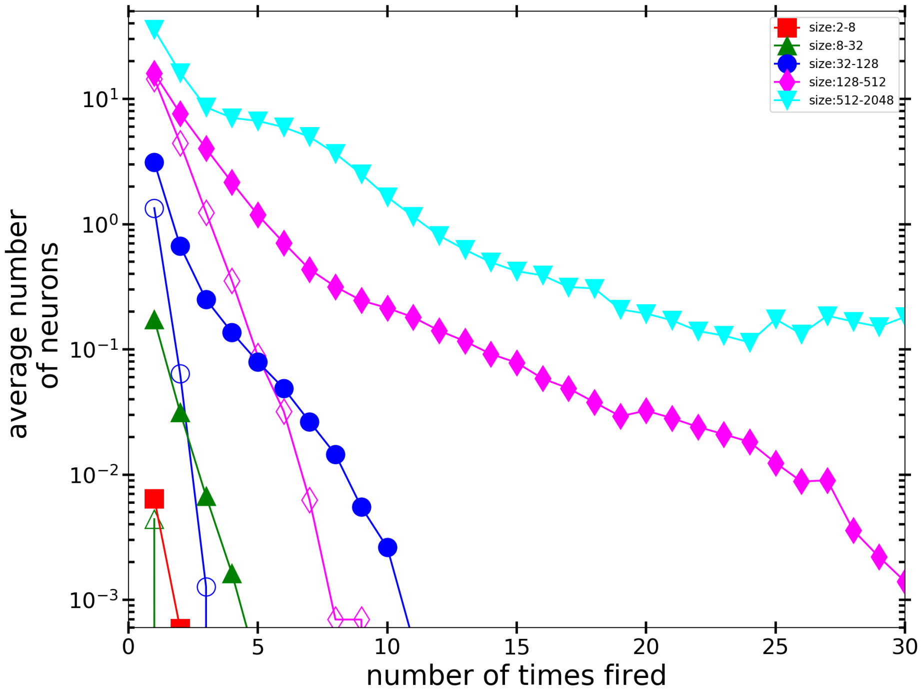

Fig. S 15: **Weakening in simulated firing time distributions.** Firing time distributions from simulations of the mouse sized system are shown with (filled markers) and without (empty markers) dynamical weakening. From this figure we can see that recurrent activation on average is largely influenced by the addition of dynamical weakening.
